# Transient Overexpression of *adh8a* Increases Allyl Alcohol Toxicity in Zebrafish Embryos

**DOI:** 10.1371/journal.pone.0090619

**Published:** 2014-03-03

**Authors:** Nils Klüver, Julia Ortmann, Heidrun Paschke, Patrick Renner, Axel P. Ritter, Stefan Scholz

**Affiliations:** 1 Department of Bioanalytical Ecotoxicology, UFZ - Helmholtz Centre for Environmental Research, Leipzig, Germany; 2 Department of Analytical Chemistry, UFZ - Helmholtz Centre for Environmental Research, Leipzig, Germany; VIB & Katholieke Universiteit Leuven, Belgium

## Abstract

Fish embryos are widely used as an alternative model to study toxicity in vertebrates. Due to their complexity, embryos are believed to more resemble an adult organism than *in vitro* cellular models. However, concerns have been raised with respect to the embryo's metabolic capacity. We recently identified allyl alcohol, an industrial chemical, to be several orders of magnitude less toxic to zebrafish embryo than to adult zebrafish (embryo LC_50_ = 478 mg/L vs. fish LC_50_ = 0.28 mg/L). Reports on mammals have indicated that allyl alcohol requires activation by alcohol dehydrogenases (Adh) to form the highly reactive and toxic metabolite acrolein, which shows similar toxicity in zebrafish embryos and adults. To identify if a limited metabolic capacity of embryos indeed can explain the low allyl alcohol sensitivity of zebrafish embryos, we compared the mRNA expression levels of Adh isoenzymes (*adh5*, *adh8a*, *adh8b* and *adhfe1*) during embryo development to that in adult fish. The greatest difference between embryo and adult fish was found for *adh8a* and *adh8b* expression. Therefore, we hypothesized that these genes might be required for allyl alcohol activation. Microinjection of *adh8a*, but not *adh8b* mRNA led to a significant increase of allyl alcohol toxicity in embryos similar to levels reported for adults (LC_50_ = 0.42 mg/L in *adh8a* mRNA-injected embryos). Furthermore, GC/MS analysis of *adh8a*-injected embryos indicated a significant decline of internal allyl alcohol concentrations from 0.23-58 ng/embryo to levels below the limit of detection (< 4.6 µg/L). Injection of neither *adh8b* nor *gfp* mRNA had an impact on internal allyl alcohol levels supporting that the increased allyl alcohol toxicity was mediated by an increase in its metabolization. These results underline the necessity to critically consider metabolic activation in the zebrafish embryo. As demonstrated here, mRNA injection is one useful approach to study the role of candidate enzymes involved in metabolization.

## Introduction

Biotransformation is an important process in the detoxification of xenobiotics. Occasionally, however, such reaction lead to products more toxic than their parent compounds. A prominent example is the metabolic activation by cytochrome P450 enzymes [1]. The limited biotransformation capacity has been a problem for the development of *in vitro* replacement methods of animal experiments used for toxicity assessment [2]. *In vitro* models, such as permanent cell lines, often exhibit a limited expression of biotransformation enzymes. This can lead to an underestimation of the toxicity of metabolically activated compounds. Therefore, supplementations of *in vitro* assays by activating reaction mixtures, for instance S9 (liver homogenate supernatant), have been applied already decades ago to compensate for the limited biotransformation capacity [3]. Alternatively, primary cells may be used for toxicity analysis, since they tend to preserve the metabolic capacity of native organs and their metabolic capacity shows greater similarities to the *in vivo* situation [4].

Fish embryos are considered as non-protected stages with regard to current animal welfare legislations and are, hence, suggested as replacement methods for the testing of animals [5], [6]. Two specific applications of regulatory interest have been extensively studied in fish embryos, particularly using zebrafish (*Danio rerio*) as a model. These approaches are the identification of environmental contaminants or drugs potentially teratogenic for humans [7]–[13] and the prediction of acute toxicity in environmental and human risk assessment [5], [10], [14]–[16].

Understanding the metabolic capacity is crucial for the development of alternative testing methods. For fish embryos, the metabolic capacity has been investigated in only a few studies. For instance, testing a set of pro-teratogens in zebrafish embryos has shown that the embryo is able to activate at least some of these substances [13]. Alderton and colleagues (2010) have investigated the metabolism of human drugs in zebrafish embryos. They found similar metabolites if compared to humans albeit the lower transformation rate indicated an overall lower biotransformation capacity [17]. Since certain compounds require metabolic activation to exert their teratogenicity, adding of a microsomal activation (MAS) mix derived from induced rat liver microsomes has been suggested to improve the capacity of fish embryos to predict acute toxicity of adult fish [8].

We recently observed that allyl alcohol, an industrial chemical, also exhibits a reduced acute lethal toxicity in zebrafish embryos [18]. If compared to toxicity levels in adult fish (0.32 mg/L in fathead minnow [19], 0.28 mg/L in zebrafish[18], allyl alcohol toxicity was only observed at about 1000-fold higher concentrations in embryos. A similar observation was made for mammalian cellular *in vitro* systems [20] and for the rainbow trout gill cell line, RTgill-W1 [21].

From mammals it is known that allyl alcohol is converted by alcohol dehydrogenases (Adhs) to the more toxic acrolein [22], [23]. Adhs are zinc-containing cytosolic enzymes and are known to metabolize alcohols to their corresponding aldehydes (e.g. ethanol to acetaldehyde). Acrolein is a soft nucleophile aldehyde with enhanced cytotoxicity, genotoxicity, and ability to impair mitochondrial function. The toxicity is most likely mediated by covalent protein modifications, formation of DNA adducts and a severe glutathione depletion [24]. In zebrafish, various Adh isoenzymes or their corresponding gene sequences, respectively, have been identified and their mRNA expression during embryogenesis has been studied [25], [26].

Our aim was to demonstrate that the discrepancy for acute lethal toxicity of allyl alcohol between fish embryos and adult fish can be attributed to a limited expression of Adh. Therefore, we analyzed expression patterns in zebrafish embryos and conducted microinjection of candidate *adh8* mRNAs in order to increase the metabolic rate. Indeed, we were able to show that the embryotoxicity of allyl alcohol is significantly and specifically increased by injection of *adh8a* mRNA. The observed decline in internal allyl alcohol concentration in embryos injected with *adh8a* supports that the increase in toxicity is caused by an enhanced metabolic conversion.

## Results

### Allyl alcohol toxicity during different stages of zebrafish embryo development

**Figure 1:**
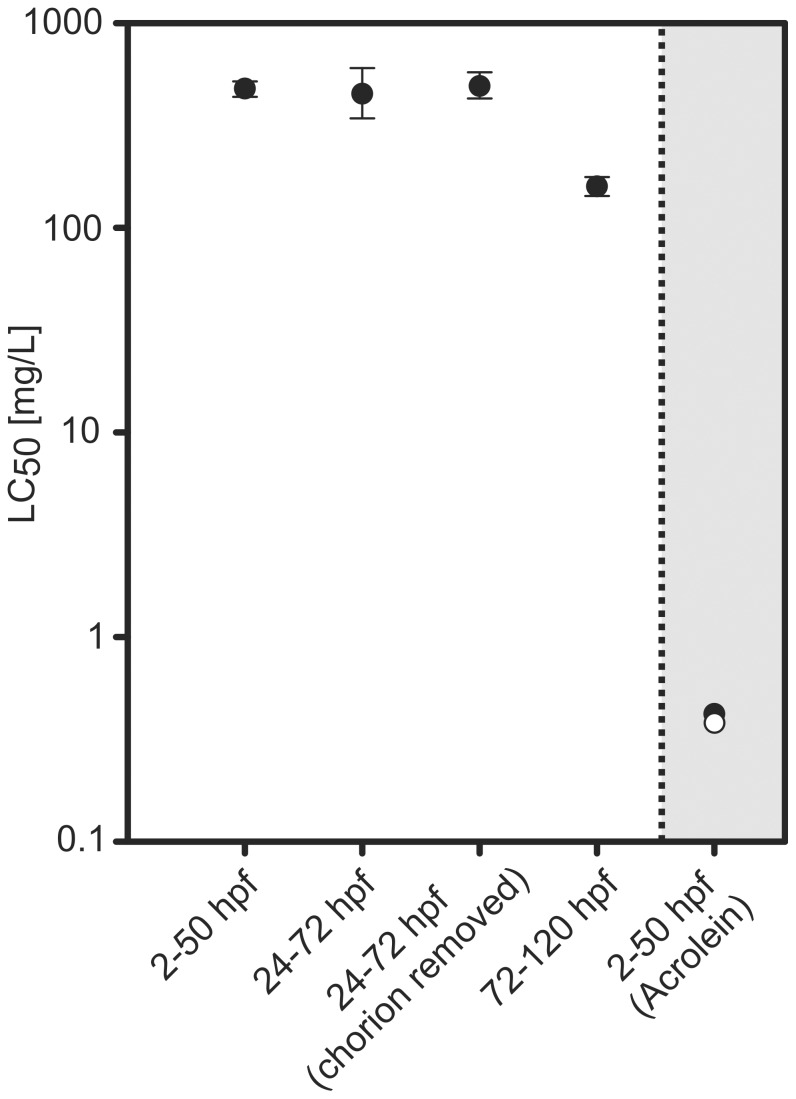
Allyl alcohol toxicity in different life stages of zebrafish. Zebrafish embryos were exposed for the indicated periods (hpf  =  hours post fertilization). For the toxicity studies we used 1 embryo per well, 10 wells per exposure concentration. Exposure from 24–72 hpf was performed with and without chorion. Error bars indicate the asymmetric 95% confidence intervals of the concentration-response curves (n = 3; for acrolein no replicates have been performed and therefore no confidence intervals have been calculated). In comparison to a previously published LC_50_ for zebrafish embryos obtained from Lammer *et al.* 2009 (open circle), acrolein toxicity based on an exposure from 2–50 hpf (n = 1) is depicted in the grey shaded area (closed symbol). Molecular weights of allyl alcohol and acrolein: 58.08 and 56.06 g/mol, respectively.

Zebrafish embryos were exposed for 48 h to various concentrations of allyl alcohol during different periods of embryonic development. For embryos exposed from 2–50 hpf, an LC_50_ of 478 mg/L (8.2 mM) was determined from concentration-response modeling (Figure 1, Figure S1 and Table S1). Exposure from 24–72 hpf with or without manually removed chorion resulted in statistically indifferent LC_50_ (451 mg/L [7.7 mM] and 492 mg/L [8.5 mM], respectively). However, a significant increase of allyl alcohol toxicity was observed for exposure from 72–120 hpf, indicated by a 3fold lower LC_50_ (157 mg/L [2.7 mM]). For acrolein, the known metabolite of allyl alcohol, an LC_50_ of 0.42 mg/L (4.28 µM) was determined for 2–50 hpf exposures (Figure 1). The approximate 1000-fold lower LC_50_ of acrolein indicated a potentially limited metabolic capacity of zebrafish embryos. No sublethal malformations were observed in exposed embryos. In toxic concentration a coagulation of the embryos was observed within 24 h of exposure (see supplemental video M1).

### Developmental expression of alcohol dehydrogenases

**Figure 2:**
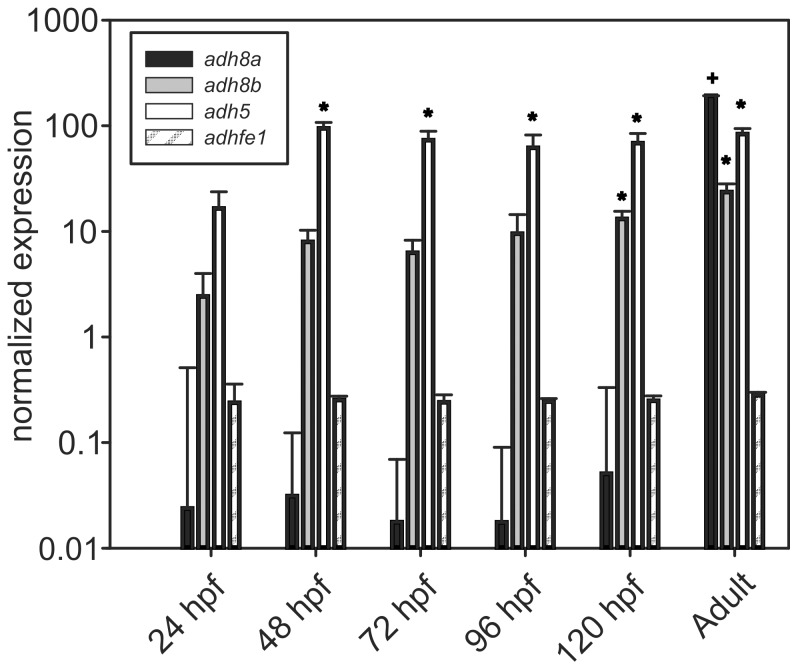
Developmental gene expression of different *alcohol dehydrogenases* (*adh*) in zebrafish embryos in comparison to adult female liver. For each developmental stage, a pool of 30 embryos has been used. Liver sample n = 3. * Significantly different to 24 hpf embryos. + Significantly different to all other stages.

In order to address the question whether a low level of Adhs in embryos may explain the lower allyl alcohol toxicity in embryos, we compared the gene expression levels of various Adhs during zebrafish development and in the adult (female) liver. Four different *adh* sequences were identified by a text search for the term “alcohol dehydrogenase” or “adh” in gene names/descriptions in the ZFIN (www.zfin.org) and ensemble database (www.ensembl.org) and an additional BLAST analysis for further homologous zebrafish cDNA sequences: *adh8a* (ENSDART00000102295), *adh8b* (ENSDART00000042766), *adh5* (ENSDART00000108825) and *adhfe1* (ENSDART00000075551). During preparation of the manuscript, Ensembl released a new zebrafish genome annotation indicating that additional Adh homologous sequences (*zgc:63568* - ENSDARG00000087262 and zgc:77938 - ENSDARG00000088366) are present in the zebrafish but these sequences could not be considered here. A phylogenetic analysis of the deduced protein sequences revealed that these new sequences are clearly Adh homologs (Figure S2). Adhfe1, in contrast, was not related to any of the other Adh sequences. qPCR experiments revealed that mRNA abundance of the *adh8a* isoenzyme exhibited a greater than 1000-fold difference between whole embryos and adult liver (Figure 2). For *adh8b*, a higher expression in 120 hpf embryos was detected, whereas in adult liver the highest *adh8a* expression levels were observed. For *adh5*, elevated levels were found in stages older than 24 hpf. No differences between embryonic stages and adult liver were observed for *adhfe1* expression (Figure 2). Based on the expression data, we hypothesized that *adh8a* and/or *adh8b* may explain the differential sensitivity of embryos and adult fish for allyl alcohol toxicity. Therefore, these two enzymes were selected for subsequent mRNA injection experiments.

### Injection of adh8 mRNAs increased allyl alcohol toxicity

**Figure 3:**
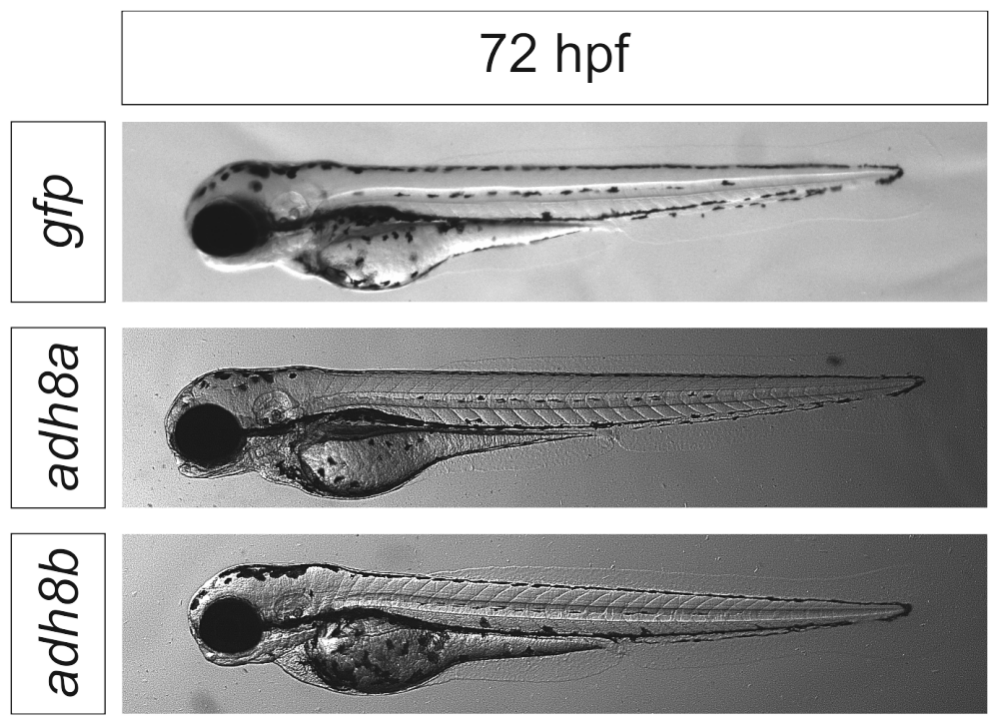
Overexpression of *adh8a* did not alter embryonic development. Lateral views of embryo at 72–2 hpf with *gfp*, *adh8a*, or *adh8b* mRNAs.

**Figure 4:**
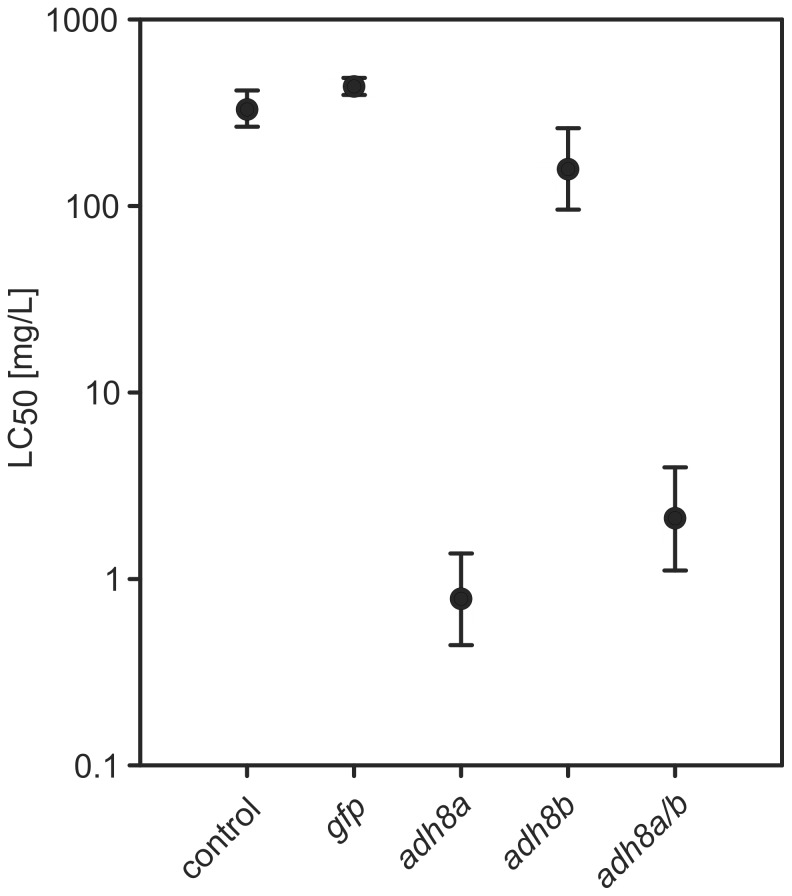
Effect of the injection of *adh8a* and *adh8b* mRNA on the LC_50_ of allyl alcohol. Error bars represent 95% asymmetric confidence intervals of LC_50_ data (see Table S1 for dose-response modeling). *gfp* mRNA was injected as an unspecific control.

In order to test the role of Adh enzymes on allyl alcohol toxicity, the corresponding mRNA was injected. We anticipated a translation into a functional protein. If a specific Adh was involved in activation of allyl alcohol, an increased biotransformation to the more potent acrolein would be expected thus leading to a reduced LC_50_. *gfp* mRNA injection was used as a control. Embryos injected with *gfp* mRNA exhibited GFP fluorescence from 2 hpf until the end of the experiment principally indicating a translation of injected mRNAs. 48 h after mRNA injection, levels of *adh8a* or *adh8b* mRNA were determined by qPCR (Table S2). The data clearly indicated that injection of mRNAs lead to a sustainable elevation of mRNA levels. The observed variability is probably related to the fact that both the injection volume (variation between approximately 0.5–2 nl) as well as the intracellular stability of the injected mRNA until 48 hpf may not be controlled precisely. However, the low variability in effect concentrations of injected embryos indicated that levels of injected mRNA have been sufficiently high to promote an effective metabolization of acrolein below the detection limit (see below). None of the injected embryos in the control experiments showed any developmental delay or malformations (Figure 3). Injection of *gfp* mRNA did not cause any significant change in the LC_50_ level and demonstrated that mRNA injection did not cause unspecific toxicity (Figure 4 and Table S1). If compared to *gfp*-injected or non-injected embryos, LC_50_ of embryos injected with *adh8a* mRNA was reduced more than 500-fold, from 478 mg/L (8.2 mM) to 0.78 mg/L (13.4µM) (Figure 4, S3A, S3B and S3C and Table S1). A slight reduction (2.5-fold) of the LC_50_ was observed for *adh8b* mRNA injections (Figure 4, S3A, S3B and S3D). A mixed (1∶1) injection of *adh8a* and *adh8b* resulted in LC_50_ similar to injections with *adh8a* alone (Figure 4, S3C and S3E and Table S1). In order to study the intracellular conversion of allyl alcohol, zebrafish embryos injected with *adh8a* and exposed to 0.5 and 1 mg/L nominal concentrations of allyl alcohol from 8–26 hpf were analyzed by GC-MS. Within this period mortality was observed (Movie S1) and hence, any effect of allyl alcohol conversion should be visible. Allyl alcohol was detected in embryos injected with *adh8b* and *gfp* mRNAs at levels from 0.23–58 pg/embryo in embryos exposed to 313–2016 µg/L (Table 1). In contrast, no allyl alcohol was found in embryos injected with *adh8a* mRNA (Table 1). Thus, Adh8a was considered as the enzyme capable to metabolically activate allyl alcohol in the zebrafish. However, we could not detect acrolein in the GC/MS chromatograms of *adh8a* injected embryos exposed to allyl alcohol.

**Table 1:** Analysis of internal allyl alcohol concentration in zebrafish embryos by GC-MS.

	Repl. 1	Repl. 2	Repl. 3
Sample	pg/embryo	pg/embryo	pg/embryo
*gfp* control	n.d.	n.d.	n.d.
*gfp* + 0.5 mg/L AAL	6	20	0.23
*gfp* 1 mg/L AAL	-	58	1
*adh8a* control	n.d.	n.d.	n.d.
*adh8a* + 0.5 mg/L AAL	0	n.d.	n.d.
*adh8a* 1mg/L AAL	-	n.d.	n.d.
*adh8b* control	-	n.d.	n.d.
*adh8b* + 0.5 mg/L AAL	-	17	1
*adh8b* 1 mg/L AAL	-	55	3
AAL measured concentrations			
	Repl. 1	Repl. 2	Repl. 3
Sample	AAL µg/L (0 h/24 h)	AAL µg/L (0 h/24 h)	AAL µg/L (0 h/24 h)
control	n.d.	n.d.	n.d.
0.5 mg/L	682/378	968/813	313/296
1 mg/L	-	2016/1922	651/470

For each replicate 50 embryos were used. AAL, allyl alcohol. n.d., not detectable.

In order to exclude that microinjection of *adh8a* mRNA generally increases chemically induced zebrafish embryo toxicity, injected embryos were also exposed to ethanol and 4-nitrophenol (4-NP). Ethanol is a known substrate of Adh and cytochrome P450 2E, CYP2E1 [25]. The metabolite acetaldehyde exhibits an about 435 (adult fish, [27]) to 720fold (zebrafish embryo, [25],) higher toxicity if compared to the parent compound with similar LC_50_ in adult fish (14.7 g/L, in fathead minnow [27]) and embryos (12 – 15 g/L, [15], [25]). Hence, the fish embryos and adult fish apparently show similar metabolic conversion capacities which may not depend on *adh8a* due to the differential expression levels in embryos and adult fish. Subsequently, injection of *adh8a* mRNA would not be anticipated to increase toxicity of ethanol. For 4-NP no evidence for metabolic activation by Adh8 is available. We determined the LC_50_ values of ethanol and 4-NP in *gfp*, *adh8a*, *adh8b*, and *adh8a*/*b* mRNA injected zebrafish embryos based on dose-response curves (Figure S4, Table S1). Indeed, we did not detect any increase in acute toxicity of ethanol or 4-NP in response to injection of *adh8* mRNA.

## Discussion

In the present study, we investigated whether the low toxicity of allyl alcohol in fish embryos may be caused by a reduced metabolic capacity of an Adh-catalyzed transformation to acrolein. In general, acute toxicity in adult fish and embryos exhibit a similar sensitivity and a high correlation [15], [16]. For allyl alcohol, however, fish embryos exhibit an about 1000fold lower toxicity (LC_50_ of 478 mg/L; 2–50 hpf) in the range of the baseline toxicity for adult fish (1919 mg/L, calculated with ECOSAR,[28]). Since in mammals allyl alcohol is known to be metabolized by Adh to the reactive acrolein [22], [23], we hypothesized that the reduced toxicity is attributable to low Adh levels in fish embryo. In order to identify potential Adh candidates for metabolism of allyl alcohol we compared expression patterns between different stages of embryonic development and adult (female) liver tissue. Different isoforms were found to be expressed already in the embryo. The *adh8a* isoform showed the lowest expression level during embryonic development but was strongly elevated in adult (female) liver. This observation was in concordance with a study on embryonic expression of *adh8a* and *adh8b* in zebrafish by Reimers *et al.* (2004a). In contrast to Reimers *et al*. (2004b), we could, however, observe an increase of *adh8b* but not of *adh8a* levels in later embryonic stages. The low levels of *adh8a* expression in whole embryos could be associated with liver organogenesis. The liver primordium is already formed around 16 hpf and liver bud formation occurs from 24–50 hpf. Hepatocytes, the functional units of the liver, differentiate from hepatoblasts thereafter. Although the liver is functional around 72 hpf and the outgrowth over the yolk surface starts, the metabolic capacity of an adult liver appears to not have been established fully at this time point [29]. We cannot directly conclude from our data that acrolein is produced in the *adh8a* overexpression studies as we could not detect acrolein in embryos by GC-MS analysis. Acrolein is highly reactive and known to form covalent glutathion, protein and DNA modifications [24]. Hence, if covalently bound to macromolecules, acrolein is most likely not detectable during the GC-MS procedure. However, we clearly observed an increased embryotoxicity of allyl alcohol in *adh8a* microinjected embryos and a reduced allyl alcohol concentration (below the limit of detection) in *adh8a* injected embryos. In contrast, in *ahd8b* and *gfp* mRNA injected control embryos allyl alcohol internal concentrations of about 0.23–58pg/embryo were detected. This observation further supports that allyl alcohol is metabolized by Adh8. The variability of allyl alcohol internal concentrations in the different control replicates (*gfp* and *adh8b* injected embryos) may partially result from variations in concentrations indicated by analytical analysis at time 0, and potential uncontrolled volatilization during the exposure and sample preparation (Table 1).Stage-specific expression analysis (*adh8a/b*) or comparison of embryonic and adult liver expression levels (*adh8a*) suggested both Adh8a and Adh8b as potential candidates for allyl alcohol activation. Since *adh5* and *adhfe1* did not exhibit any differential expression we concluded that they are probably not involved in allyl alcohol metabolism. Adh8a and Adh8b share 86% amino acid similarity, but exhibit different substrate specificities. Adh8a has a low activity toward long chain alkyl alcohols and greater activity toward ethanol, whereas Adh8b is known to prefer longer-chain alcohols [26]. In order to test which of these two isoforms may be involved in allyl alcohol activation, we used *adh8* mRNA injection into the 1-cell embryonic stage. Only *adh8a* overexpression led to a strong increase in allyl alcohol toxicity resulting in LC_50_ levels only two-fold different to adult fish. We also cannot exclude that the weak effect of *adh8b* injection on allyl alcohol toxicity may have been caused by translation into a non-functional protein. Furthermore, two other isoforms of Adh (*zgc:63568* - ENSDARG00000087262 and zgc:77938 - ENSDARG00000088366) could principally be involved in allyl alcohol metabolism as well. However, their expression patterns have not been analyzed nor is anything known about their potential substrate specificity.

The effect of *adh8a* mRNA injection cannot be attributed to an unspecific increase in sensitivity of embryos to exposure with chemicals. First, injection of mRNAs did not affect zebrafish embryonic or larval development. Second, exposure of injected embryos to two chemicals (ethanol and 4-NP) that are known to exhibit similar toxicity in embryos and adults did not provoke enhanced toxicity. Ethanol is a known substrate of Adhs and cytochrome P450- 2E [26]. Since it does not exhibit a differential toxicity in adult fish and embryos and hence, Adh8a activity in embryos may not limit the ethanol conversion and its toxicity. Hence, metabolic capacities of embryos and adults for ethanol are likely to be similar. Co-exposures of ethanol with chemical inhibitors of ethanol-metabolizing enzymes in zebrafish embryos have shown no increase in embryo lethality, although a significant increase in the occurrence of pericardial edema was detected [30]. This is in line with our observation that overexpression of *adh8* mRNAs did not affect ethanol embryotoxicity. 4-NP is not a potential substrate of Adh nor has it been recorded to exhibit reduced toxicity in embryos (LC_50_zebrafish embryo_  =  12.0 mg/L, unpublished data; LC_50_fathead minnow_  =  44.8 mg/L, [27]).

Differences of compound uptake between embryos and adults could principally also result in a differential sensitivity. In both embryos and adults exposure internal concentrations are mediated by a partition equilibrium with the concentration in the test medium. In contrast to adults, the chorion could provide an additional barrier resulting in lower internal concentration and toxicity. However, so far the chorion has been shown to mainly represent a physical barrier for organic chemicals with high molecular weight [31]. Therefore, the analysis of allyl alcohol toxicity, the effect of *adh8a* injection and the corresponding internal concentrations strongly support that the reduced toxicity of allyl alcohol in fish embryos is caused by a weak metabolization if compared to adult fish.

In summary, we demonstrated that allyl alcohol toxicity is dependent on the *adh* mRNA/Adh protein abundance and that at least one isoform, i.e. Adh8a, is greatly mediating allyl alcohol toxicity. It is likely that Adh8a causes an increased metabolic conversion to its primary metabolite, acrolein because *adh8a* mRNA injected embryos exhibit a similar LC_50_ as observed for adults and for acrolein exposure. Further evidence for metabolization of allyl alcohol to acrolein might be provided by analysis of covalent modifications. This, however, would require the development of appropriate specific and sensitive methods to detect acrolein adducts in zebrafish embryos.

The injection of mRNA was shown to be a powerful approach to test the role of specific enzymes in metabolic activation (and for deactivation probably as well). Furthermore, it has confirmed the crucial role of biotransformation in the development of alternative testing methods. However, due to its specificity, microinjection cannot serve as a general approach to improve the metabolic capacity of fish embryos. Potential alternatives may be the use of induced rat liver microsomes or S9 mix. Supplements have frequently been used in cell cultures but also in teratogenicity assays using amphibian or fish embryos [8], [32]. In fish embryos, teratogenic effects have been enhanced or were only observed when embryos were exposed for 1 h (2–3 hpf) to rat liver microsomes. We tried to develop protocols that allow a continuous supplement of exposure media with S9 mix or microsomes. However, long-term supplementation resulted in a high background toxicity (data not published). Therefore, future improvements of the fish embryo test may include pre-exposure to metabolic activation mixture and extraction of metabolites. Alternatively, potential metabolites may be identified by exposure of the parent compound to an S9 mix, microsomes and/or by computational approaches [33]–[35] (OECD toolbox freely available at www.oecd.org). Potential metabolites may then be tested in addition to the parent compounds in order to reduce the risk of under-prediction of toxicity.

## Materials and Methods

### Ethics Statement

All zebrafish husbandry and experimental procedures were performed in accordance with the German animal protection standards and were approved by the Government of Saxony, Landesdirektion Leipzig, Germany (Aktenzeichen 75-9185.64). Based on the *Guidelines on the protection of experimental animals* by the Council of Europe, Directive 2010/63/EU, which allows zebrafish embryos to be used up to the moment of independent feeding (approximately 5 days after fertilization). Because embryos used here were no more than 4 days old, no license is required by Council of Europe (1986), Directive 2010/63/EU or the local authority.

### Fish maintenance

Two zebrafish strains – WiK and UFZ-OBI - have been used. The WiK zebrafish strain was obtained from the Max Planck Institute for Developmental Biology (Tuebingen, Germany) and has been kept for several generations at the UFZ. The OBI strain was established from a broodstock of a local supplier (OBI hardware store, Leipzig, Germany) and the F1 generation has been used for production of embryos. Both strains exhibit similar performance with respect to sensitivity to allyl alcohol (see Table S1) and other chemicals (data not shown). Fish were maintained in local tap water (conductivity 540–560 µS/cm, water hardness 2–3 mM divalent ions, pH 7–8, oxygen saturation 87–91%) in a circulating tank system with central biological filter unit. About 1% of the tank water was replaced by fresh dechlorinated tap water per hour. Nitrate (< 2.5 mg/L), nitrite (≤ 0.025 mg/L) and ammonium (≤ 0.6 mg/L) concentrations were checked weekly. Water temperature was adjusted to 26±1°C and monitored daily. A light cycle of 14∶10 h (light:dark) was applied to stimulate spawning of the fish. Twenty-five to 30 fish were kept per 14 L tank with random sex distribution. The age of fish in spawning tanks was between 6 and 18 months. Fish were fed daily, twice with live *Artemia* and once with commercial flake food (Tetra, Melle, Germany). Spawning was stimulated by placing glass trays covered with a 3 mm mesh and artificial plants into the breeding tanks the evening before fertilized eggs were needed. Eggs were collected from spawning trays within one hour after the onset of light and poured into 100 µm mesh. After rinsing them several times with tank water, embryos were transferred into a crystallization dish and incubated at 26±1°C.

### Zebrafish embryo test

The zebrafish embryo test was performed with few modifications according to a standard operation procedure and a static exposure setup without renewal of exposure solutions (ISO 15088, 2006). The following chemicals were used: Allyl alcohol (CAS 107-18-6, purity 99.8%, Sigma-Aldrich, Deisenhofen, Germany), acrolein (CAS 107-02-8, purity ≥99.0%, Sigma-Aldrich, Deisenhofen, Germany), 4-Nitrophenol (CAS 100-02-7, PESTANAL, analytical standard, Sigma-Aldrich, Deisenhofen, Germany), and ethanol (CAS 64-17-5, purity ≥ 99.5%, Carl Roth, Germany). Stock solutions were prepared for each experiment by adding the appropriate amount for the highest test concentration to the exposure media (294.0 mg/L CaCl_2_*2H_2_O, 123.3 mg/L MgSO_4_*7H_2_O, 64.7 mg/L NaHCO_3_, 5.7 mg/L KCl). The solutions were prepared one day before the experiment in 100 ml closed graduated flasks and were stirred over night for approximately 14 h. Lower concentrations were obtained by serial dilutions. Fertilized eggs (embryos at 4–32 cell stage) were transferred by a pipette to 24-well plates (1 embryo per well, 10 wells per exposure concentration, Cellstar Greiner Bio-One, Frickenhausen, Germany) or crystallization glass dishes (80 mm diameter, 10–20 embryos per vial and 30 ml of exposure medium), respectively. Exposures were performed in independent triplicates on different days for 48 h from 2-50 hpf (hours post fertilization), 24–72 hpf, and 72–120 hpf. For the 24–72 hpf exposure, part of the embryos were manually dechorionated using forceps. In order to detect and remove embryos that have been mechanically injured by microinjections, exposure in microinjection experiment did not start before 8 hpf and was continued until 26 and 56 hpf, respectively. At either 2, 8, 24 or 72 hpf the medium was completely removed and replaced by exposure media containing different concentrations of allyl alcohol (2 ml per well or 30 ml per crystallization dish, respectively). The Henry's law coefficients of the test chemicals (allyl alcohol: 4.99*10^−6^, ethanol: 5.62*10^−6^, 4-nitrophenol: 4.15*10^−10^, acrolein: 1.22*10^−4^; atm*m^3^/mol) indicate a weak volatility for ethanol, allyl alcohol and acrolein. Therefore, in order to reduce potential evaporation, 24-well plates were covered with an adhesive foil; crystallization dishes were covered with a household plastic wrap and watch glasses. For comparison of the LC_50_ at different stages and the effect of dechorionation, exposure was performed in 24-well plates. For gene expression analysis and microinjection experiments crystallization dishes were used for exposure.

After 48 h of exposure referring to 50, 72 or 120 hpf stages, the number of dead embryos was counted. Dead embryos were detected according to the criteria given in the ISO standard [36]; i.e., either coagulation, missing heartbeat, failure to develop somites or a lack of detachment of the tail-bud from the yolk sac were considered as indicators of lethality.

Allyl alcohol stability has not been analyzed in this study but data were available from the CEllSens study [37] with 24-well plates [18]. In this study measured concentrations (0 h) referred to about 80% of nominal concentrations. Within 48 h of exposure a decline to about 28% of the nominal concentration was observed. Since (1) these changes in measured exposure concentrations were well below one order of magnitude (47% of the nominal concentration based on the geometric mean of measured concentrations), (2) the determination of the real effective concentration is difficult due to the decline and (3) changes in toxicity induced by mRNA injection span several order of magnitude, we considered the use of nominal exposure concentrations as appropriate.

### Time-lapse recording

For time lapse recording of zebrafish embryos were performed using a Leica DMI4000 B inverted microscope (Leica, Wetzlar, Germany), equipped with a digital camera (DFC 350FX; Leica) and 5× objective (HCX PL FLUOTAR, 5.0×0.15 DRY). adh8a mRNA injected were exposed to 5mg/L allyl alcohol in 24-well plates (2 embryos per 2 ml). A constant temperature of 26°C was maintained with a heated stage insert. Embryos were imaged by bright filed every 20 min for 20 h. Images were analyzed using LAS AF software (Leica, Wetzlar, Germany). Video was exported using 3 frames per second.

### Quantitative real-time PCR (qPCR)

For qPCR, total RNA was isolated from 30 embryos per replicate/treatment and the liver of a female adult fish using Trizol (Invitrogen, Karlsruhe, Germany) according to the manufacturer's instructions. RNA integrity was checked by agarose gel electrophoresis. Potential contamination with genomic DNA was removed by treatment with DNAse I (Roche, Grenzach, Germany) for 15 min at 25°C prior to performing the cDNA synthesis. cDNA was synthesized from 2 µg of total RNA using the Fermentas RevertAid kit (Fermentas, Bad Leon-Roth, Germany) and random hexamer primers. cDNA was diluted 4× with ddH_2_O. Primers were designed to span introns with the software Beacon Designer 7 (PREMIER Biosoft International, CA, USA). For Primer Sequences see Table S3. The qPCR analysis was performed for two (injected embryos) and three (time course of mRNA levels) independent biological replicates. qPCR was carried out using a StepOnePlus PCR System (Applied Biosystems, Darmstadt, Germany) and we used the SensiMix SYBR with ROX as passive reference (Bioline). Each run was performed with three technical replicates per sample. The reaction conditions: 95°C 15 s, 53°C 20 s, and 72°C 20 s). Expression was normalized against *ef1a* as reference gene and the normalized relative expression levels were determined by the ΔΔCt method [38].

### Microinjection

Injection needles were pulled from borosilicate glass capillary tubes with filament (GC100F-10; Warner Instruments, LLC, Hamden, CT) using a Narishige micropipette puller (Tokyo, Japan). Embryos were injected with the Eppendorf FemtoJet (Hamburg, Germany) through the chorion into the yolk compartment at the one-cell stage.

Plasmids with either the cDNA sequence of *adh8a* or *adh8b* were purchased from ImaGenes (Berlin, Germany, clone numbers IRBOp991G0334D for *adh8a* and IRBOp991F02101D for *adh8b*). The GFP open reading frame was cloned into the pCS2p+ Vector. For synthesis of capped mRNAs, *adh8* plasmids were cut with *XhoI* and pCS2P+GFP was cut with *KpnI*. 5′ capped mRNA was synthesized with the mMessage Machine SP6 Kit (Life Technologies/Ambion, Darmstadt, Germany) according to the manufacturer's instructions. Injected mRNA concentration was 20–50 ng/µl. Phenol red was added to the RNA samples before injection (0.1% final concentration) in order to allow tracking of successful injections.

### Quantification of internal allyl alcohol concentration

50 injected embryos were exposed from 8 hpf to 26 hpf with 0.5 or 1 mg/L allyl alcohol. At 26 hpf embryos were manually dechorionated using forceps and transferred into fresh 20 ml glass vials. The exposure medium was removed and embryos were rinsed twice with 2 ml ISO water for 30 sec, transferred into GC-MS headspace vials (Agilent Technologies, Böblingen, Germany). After removal of remaining fluid by using a microliter pipette the vials were closed and embryos were frozen at −20°C for 10 min. Subsequently, embryos were homogenized by 5 min sonication in an ultrasonic bath (Sonorex RK 512 H; Bandelin Electronic, 35 kHz). Chemical analyses were performed immediately after sonication using an Agilent 7890A gas chromatograph with headspace sampler G1888 (Agilent Technologies, Böblingen, Germany). The headspace oven temperature for equilibration of samples was set to 48°C for 10 min. The compound separation was performed using a capillary column (60 m×0.25 i.d.) with a nonpolar stationary phase HP1 (thickness 1.00 µm). Ultra high purity helium was used as carrier gas. Mass spectra were derived with Agilent 5975C inert XL MSD and Triple Axis Detector (Agilent Technologies,) in electron impact ionization mode 70eV. The detector conditions were: ion source temperature 230°C and quadrupole temperature 150°C. The column oven temperature program involved an initial temperature of 45°C for 1 min, an increase at 15°C/min to 150°C (held for 2 min), then an increase at 65°C/min to 250°C (held 0.5 min). The mass spectrometer was operated in selected ion monitoring (SIM) mode for calibration and analyte quantification in samples.

For the allyl alcohol calibration the following concentrations were used: 8.5; 42.5; 85; 425; 850 and 1700 µg/L. 5 µl of the respected concentration were transferred into a headspace vial and directly used for HS-GC-MS analysis. The calibration curve was generated based on peak areas. Exposure concentrations were determined for each replicate at the start of the exposure.

### Data analysis and statistics

The LC_50_ values ± asymmetric 95% confidence intervals were calculated using the software JMP (SAS, Cary, NC) using the Hill-slope equation for modeling of concentration-response curves:
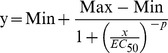



Min and Max were set to 0 and 100%, respectively. *x* and *y* represented the nominal exposure concentration and the survival rate (percentage).

Statistical significance analysis was performed by ANOVA and Dunnett's test. Residues had been tested for normality distribution prior to significance analysis. In case of non-homogenous variances (*adh8a* expression data) an ANOVA with Dunnet's T3 test was applied. Statistical significance of LC_50_ data was assessed based on the 95% confidence intervals. Data with overlapping confidence intervals were considered not statistically significant different.

## Supporting Information

Figure S1 **Stage dependent allyl alcohol concentration-response curves of zebrafish embryos.** Concentration-response curves were modeled based on the Hill-slope equation. For the toxicity studies we used 1 embryo per well, 10 wells per exposure concentration. A. Allyl alcohol exposure from 2-50 hpf. B. Allyl alcohol exposure from 24-72 hpf. C. Allyl alcohol exposure from 24-72 hpf with chorion manually removed at 24 hpf. D. Allyl alcohol exposure from 72-120 hpf. E. Acrolein exposure from 2-50 hpf. Please note that only one replicate was performed for acrolein exposure in order to confirm the published LC_50_ [15].
(TIF)

Figure S2 **Phylogenetic tree of vertebrate Adh proteins**. Zebrafish (Dr), human (Hs) and mice (Mm). Phylogeny was constructed by the neighbor-joining method. Numbers on each branch are bootstrap values from 1000 replicates. Scale bar = 0.05 substitutions per site. Zebrafish Adh related proteins are grey-shaded. Accession numbers: **Hs** ADH1a NP_000658, ADH1B NP_000659, ADH4 NP_000661, ADH5 CAG38730, ADH6 AAH39065, ADH7 AAB38424. **Mm** Adh1 NP_031435, Adh6a NP_081221, Adh4 NP_036126, Adh5 AAH62879, Adh6b XP_003688830, Adh7 NP_033756. **Dr** zgc:63568 NP_956749, zgc:77938 AAH65900, Adh5 AAH67170, Adh8a AAI65868, Adh8b NP_982285, Adhfe1 AAH66529.
(TIF)

Figure S3 **Allyl alcohol concentration-response curves of** ***gfp*** **,** ***adh8a*** **, and** ***adh8b*** **mRNA injected and non-injected zebrafish embryos.** Allyl alcohol exposure was performed from 8-56 hpf and mortality was measured (n = 3, see also Table S1). A. non-injected (control). B. *gfp* mRNA injected (injection control) C. *adh8a* mRNA injected. D. *adh8b* mRNA injected. E. *adh8a/adh8b* mRNA injected.
(TIF)

Figure S4 **4-Nitrophenol and ethanol concentration-response curves of** ***gfp*** **,** ***adh8a*** **, and** ***adh8b*** **mRNA injected zebrafish embryos.** Exposures were performed in crystallization glass dishes with 10-20 embryos per vial and 30 ml of exposure medium. (A-D) 4-Nitrophenol concentrations are given in mg/L. A. *gfp* mRNA injected (injection control) B. *adh8a* mRNA injected. C. *adh8b* mRNA injected. D. *adh8a/adh8b* mRNA injected. (E-H) Ethanol concentrations are given in mM. E. *gfp* mRNA injected (injection control) F. *adh8a* mRNA injected. G. *adh8b* mRNA injected. H. *adh8a/adh8b* mRNA injected.
(TIF)

Table S1 **Parameter of concentration-response modeling (Hill-slope equation) and summary of LC_50_ data.** For the toxicity studies in 24-wells we used 1 embryo per well, 10 wells per exposure concentration. For exposures in crystallization glass dishes we used 10-20 embryos per vial. Exposures were performed in independent triplicates. 24WP = 24well plates, CD  =  crystallization dish (w  =  with, w/o  =  without, h  =  hatched).
(XLS)

Table S2 **Gene expression levels of** ***adh8a*** **and** ***adh8b*** **in 56 hpf embryos following an injection of either** ***gfp*** **,** ***adh8a*** **or** ***adh8b*** **mRNA in the 1-8 cell stage.** Expression was analyzed by qPCR for two replicates. For each replicate 30 embryos were pooled. Fold changes are based on normalized expression using *ef1a* as reference gene. Primers of *adh8b* had a high sequence similarity to adh8a (adh8b-F1: 4 out of 21 nucleotides were different; adh8b-R1: 4 out of 18 nucleotides were different).
(XLS)

Table S3 **Primers used in qPCR.**
(XLS)

Movie S1 **Time laps recording of allyl alcohol toxicity of** ***adh8a*** **injected embryos.** Embryos were injected with *adh8a* mRNA and exposed to 5 mg/L allyl alcohol in 24-wells (2 embryos per 2 ml). The movie shows the induced allyl alcohol toxicity through *adh8a* mRNA overexpression. Coagulation of the embryos was observed after 3-9 h allyl alcohol exposure. Overexpression of *adh8a* alone did not result in adverse development effects (Figure 4).
(AVI)

## References

[1] Goldstein JA, Faletto MB (1993) Advances in mechanisms of activation and deactivation of environmental chemicals. Environ Health Perspect 100: 169–176. 8354165 10.1289/ehp.93100169 PMC1519589

[2] Spielmann H, Seiler A, Bremer S, Hareng L, Hartung T, et al (2006) The practical application of three validated in vitro embryotoxicity tests. The report and recommendations of an ECVAM/ZEBET workshop (ECVAM workshop 57). Altern Lab Anim 34: 527–538. 17121476 10.1177/026119290603400504

[3] Borenfreund E, Puerner JA (1987) Short-term quantitative in vitro cytotoxicity assay involving an S-9 activating system. Cancer Lett 34: 243–248. 3828978 10.1016/0304-3835(87)90173-x

[4] Flouriot G, Vaillant C, Salbert G, Pelissero C, Guiraud JM, et al (1993) Monolayer and aggregate cultures of rainbow trout hepatocytes: long-term and stable liver-specific expression in aggregates. J Cell Sci 105 (Pt 2): 407–416. 10.1242/jcs.105.2.407 8408274

[5] Embry MR, Belanger SE, Braunbeck TA, Galay-Burgos M, Halder M, et al (2010) The fish embryo toxicity test as an animal alternative method in hazard and risk assessment and scientific research. Aquat Toxicol 97: 79–87. 20061034 10.1016/j.aquatox.2009.12.008

[6] Strahle U, Scholz S, Geisler R, Greiner P, Hollert H, et al (2012) Zebrafish embryos as an alternative to animal experiments-A commentary on the definition of the onset of protected life stages in animal welfare regulations. Reprod Toxicol 33: 128–132. 21726626 10.1016/j.reprotox.2011.06.121

[7] Brannen KC, Panzica-Kelly JM, Danberry TL, Augustine-Rauch KA (2010) Development of a zebrafish embryo teratogenicity assay and quantitative prediction model. Birth Defects Res B Dev Reprod Toxicol 89: 66–77. 20166227 10.1002/bdrb.20223

[8] Busquet F, Nagel R, von Landenberg F, Mueller SO, Huebler N, et al (2008) Development of a new screening assay to identify proteratogenic substances using zebrafish danio rerio embryo combined with an exogenous mammalian metabolic activation system (mDarT). Toxicol Sci 104: 177–188. 18375544 10.1093/toxsci/kfn065

[9] Gustafson AL, Stedman DB, Ball J, Hillegass JM, Flood A, et al.. (2012) Inter-laboratory assessment of a harmonized zebrafish developmental toxicology assay – Progress report on phase I. Reproductive Toxicology in press. 10.1016/j.reprotox.2011.12.004 22210281

[10] Padilla S, Corum D, Padnos B, Hunter DL, Beam A, et al.. (2012) Zebrafish Developmental Screening of the ToxCast(TM) Phase 1 Chemical Library. Reproductive Toxicology in press. 10.1016/j.reprotox.2011.10.018 22182468

[11] Selderslaghs IW, Van Rompay AR, De Coen W, Witters HE (2009) Development of a screening assay to identify teratogenic and embryotoxic chemicals using the zebrafish embryo. Reprod Toxicol 28: 308–320. 19447169 10.1016/j.reprotox.2009.05.004

[12] Selderslaghs IWT, Blust R, Witters HE (2012) Feasibility study of the zebrafish assay as an alternative method to screen for developmental toxicity and embryotoxicity using a training set of 27 compounds. Reproductive Toxicology in press. 10.1016/j.reprotox.2011.08.003 21871558

[13] Weigt S, Huebler N, Strecker R, Braunbeck T, Broschard TH (2011) Zebrafish (Danio rerio) embryos as a model for testing proteratogens. Toxicology 281: 25–36. 21237239 10.1016/j.tox.2011.01.004

[14] Ali S, Mil HGJv, Richardson MK (2011) Large-Scale Assessment of the Zebrafish Embryo as a Possible Predictive Model in Toxicity Testing. PLoS ONE 6: e21076. 21738604 10.1371/journal.pone.0021076 PMC3125172

[15] Lammer E, Carr GJ, Wendler K, Rawlings JM, Belanger SE, et al (2009) Is the fish embryo toxicity test (FET) with the zebrafish (Danio rerio) a potential alternative for the fish acute toxicity test? Comp Biochem Physiol C Toxicol Pharmacol 149: 196–209. 19095081 10.1016/j.cbpc.2008.11.006

[16] Nagel R, Bresch H, Caspers N, Hansen PD, Markert M, et al (1991) Effect of 3,4-dichloroaniline on the early life stages of the zebrafish (Brachydanio rerio): results of a comparative laboratory study. Ecotoxicol Environ Saf 21: 157–164. 2065628 10.1016/0147-6513(91)90017-j

[17] Alderton W, Berghmans S, Butler P, Chassaing H, Fleming A, et al (2010) Accumulation and metabolism of drugs and CYP probe substrates in zebrafish larvae. Xenobiotica 40: 547–557. 20528625 10.3109/00498254.2010.493960

[18] Knobel M, Busser FJ, Rico-Rico A, Kramer NI, Hermens JL, et al (2012) Predicting adult fish acute lethality with the zebrafish embryo: relevance of test duration, endpoints, compound properties, and exposure concentration analysis. Environ Sci Technol 46: 9690–9700. 22835061 10.1021/es301729q

[19] Geiger DLB, L. T.; Call, D J. (1990) Acute Toxicities of Organic Chemicals to Fathead Minnows (Pimephales promelas): Center for Lake Superior Environmental Studies, University of Wisconsin, Superior, WI, USA.

[20] Kramer NI, Hermens JL, Schirmer K (2009) The influence of modes of action and physicochemical properties of chemicals on the correlation between in vitro and acute fish toxicity data. Toxicol In Vitro 23: 1372–1379. 19651202 10.1016/j.tiv.2009.07.029

[21] Tanneberger K, Knobel M, Busser FJ, Sinnige TL, Hermens JL, et al (2013) Predicting fish acute toxicity using a fish gill cell line-based toxicity assay. Environ Sci Technol 47: 1110–1119. 23227966 10.1021/es303505z

[22] Atzori L, Dore M, Congiu L (1989) Aspects of allyl alcohol toxicity. Drug Metabol Drug Interact 7: 295–319. 2489200

[23] Auerbach SS, Mahler J, Travlos GS, Irwin RD (2008) A comparative 90-day toxicity study of allyl acetate, allyl alcohol and acrolein. Toxicology 253: 79–88. 18817840 10.1016/j.tox.2008.08.014 PMC2665691

[24] Cai J, Bhatnagar A, Pierce WM Jr (2009) Protein modification by acrolein: formation and stability of cysteine adducts. Chem Res Toxicol 22: 708–716. 19231900 10.1021/tx800465m PMC2929760

[25] Reimers MJ, Flockton AR, Tanguay RL (2004) Ethanol- and acetaldehyde-mediated developmental toxicity in zebrafish. Neurotoxicol Teratol 26: 769–781. 15451041 10.1016/j.ntt.2004.06.012

[26] Reimers MJ, Hahn ME, Tanguay RL (2004) Two zebrafish alcohol dehydrogenases share common ancestry with mammalian class I, II, IV, and V alcohol dehydrogenase genes but have distinct functional characteristics. J Biol Chem 279: 38303–38312. 15231826 10.1074/jbc.M401165200 PMC3261772

[27] Russom CL, Bradbury SP, Broderius SJ, Hammermeister DE, Drummond RA (1997) Predicting modes of toxic action from chemical structure: acute toxicity in the fathead minnow (Pimephales promelas). Environmental Toxicology and Chemistry 16: 948–967. 10.1002/etc.2249 23733666

[28] Clements RG, Nabholz JV (1994) ECOSAR: a computer program and user's guide for estimating the ecotoxicity of industrial chemicals based on structure activity relationships. EPA-748-R-93-002, US Environmental Protection Agency, Office of Pollution Prevention and Toxics 7403, Washington, DC.

[29] Tao T, Peng J (2009) Liver development in zebrafish (Danio rerio). J Genet Genomics 36: 325–334. 19539242 10.1016/S1673-8527(08)60121-6

[30] Reimers MJ, La Du JK, Periera CB, Giovanini J, Tanguay RL (2006) Ethanol-dependent toxicity in zebrafish is partially attenuated by antioxidants. Neurotoxicol Teratol 28: 497–508. 16904866 10.1016/j.ntt.2006.05.007

[31] Henn K, Braunbeck T (2011) Dechorionation as a tool to improve the fish embryo toxicity test (FET) with the zebrafish (Danio rerio). Comp Biochem Physiol C Toxicol Pharmacol 153: 91–98. 20869464 10.1016/j.cbpc.2010.09.003

[32] Hoke RA, Ankley GT (2005) Application of frog embryo teratogenesis assay-Xenopus to ecological risk assessment. Environ Toxicol Chem 24: 2677–2690. 16268171 10.1897/04-506r.1

[33] Cowan-Ellsberry CE, Dyer SD, Erhardt S, Bernhard MJ, Roe AL, et al (2008) Approach for extrapolating in vitro metabolism data to refine bioconcentration factor estimates. Chemosphere 70: 1804–1817. 17904615 10.1016/j.chemosphere.2007.08.030

[34] Escher BI, Cowan-Ellsberry CE, Dyer S, Embry MR, Erhardt S, et al (2011) Protein and lipid binding parameters in rainbow trout (Oncorhynchus mykiss) blood and liver fractions to extrapolate from an in vitro metabolic degradation assay to in vivo bioaccumulation potential of hydrophobic organic chemicals. Chem Res Toxicol 24: 1134–1143. 21604782 10.1021/tx200114y

[35] Madden JC, Cronin MT (2006) Structure-based methods for the prediction of drug metabolism. Expert Opin Drug Metab Toxicol 2: 545–557. 16859403 10.1517/17425255.2.4.545

[36] ISO_15088 (2006) Water quality – determination of the acute toxicity of waste water to zebrafish eggs (Danio rerio). ISO 15088: 2007 (E)..

[37] Schirmer K, Tanneberger K, Kramer NI, Volker D, Scholz S, et al (2008) Developing a list of reference chemicals for testing alternatives to whole fish toxicity tests. Aquat Toxicol 90: 128–137. 18829120 10.1016/j.aquatox.2008.08.005

[38] Pfaffl MW (2001) A new mathematical model for relative quantification in real-time RT-PCR. Nucleic Acids Res 29: e45. 11328886 10.1093/nar/29.9.e45 PMC55695

