# Supplementary figures and images for: Transient Overexpression of adh8a Increases Allyl Alcohol Toxicity in Zebrafish Embryos
Source: PLoS One. 2014 Mar 3;9(3):e90619. doi: 10.1371/journal.pone.0090619 (PMC3940891; doi:10.1371/journal.pone.0090619)

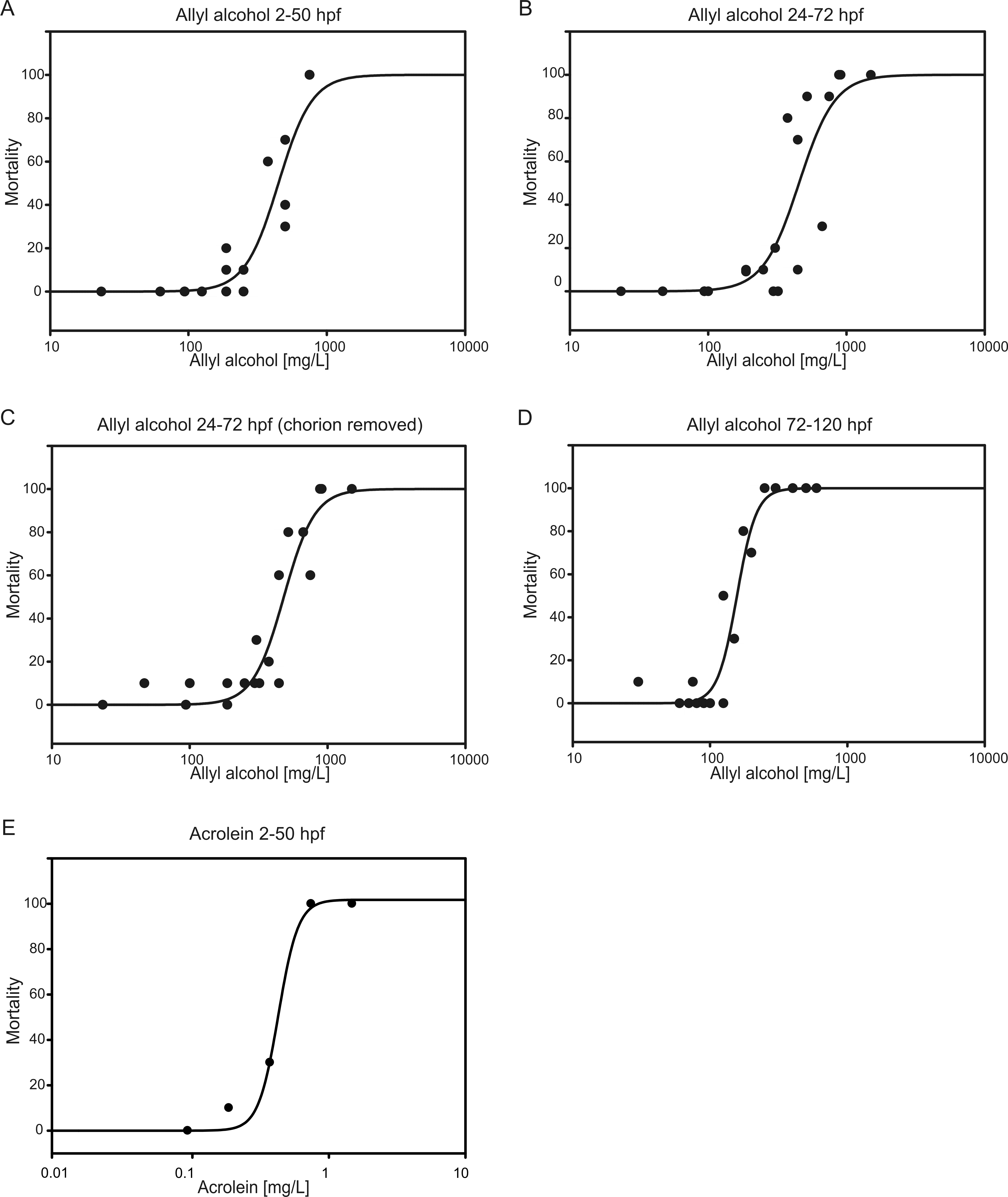

Supplement: Figure S1 — Stage dependent allyl alcohol concentration-response curves of zebrafish embryos. Concentration-response curves were modeled based on the Hill-slope equation. For the toxicity studies we used 1 embryo per well, 10 wells per exposure concentration. A. Allyl alcohol exposure from 2-50 hpf. B. Allyl alcohol exposure from 24-72 hpf. C. Allyl alcohol exposure from 24-72 hpf with chorion manually removed at 24 hpf. D. Allyl alcohol exposure from 72-120 hpf. E. Acrolein exposure from 2-50 hpf. Please note that only one replicate was performed for acrolein exposure in order to confirm the published LC50 [15]. (TIF) [file pone.0090619.s001.tif]

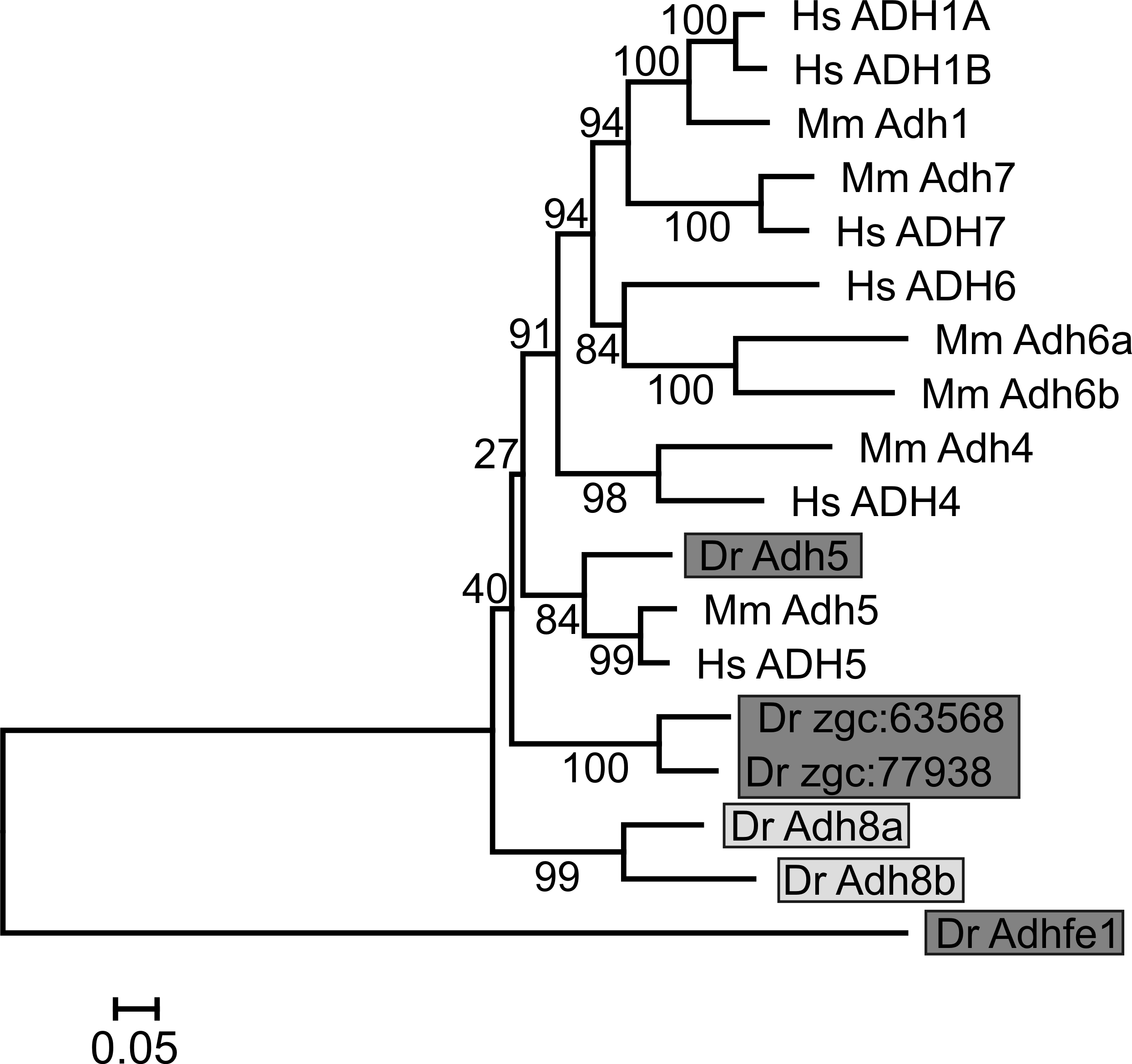

Supplement: Figure S2 — Phylogenetic tree of vertebrate Adh proteins. Zebrafish (Dr), human (Hs) and mice (Mm). Phylogeny was constructed by the neighbor-joining method. Numbers on each branch are bootstrap values from 1000 replicates. Scale bar = 0.05 substitutions per site. Zebrafish Adh related proteins are grey-shaded. Accession numbers: Hs ADH1a NP_000658, ADH1B NP_000659, ADH4 NP_000661, ADH5 CAG38730, ADH6 AAH39065, ADH7 AAB38424. Mm Adh1 NP_031435, Adh6a NP_081221, Adh4 NP_036126, Adh5 AAH62879, Adh6b XP_003688830, Adh7 NP_033756. Dr zgc:63568 NP_956749, zgc:77938 AAH65900, Adh5 AAH67170, Adh8a AAI65868, Adh8b NP_982285, Adhfe1 AAH66529. (TIF) [file pone.0090619.s002.tif]

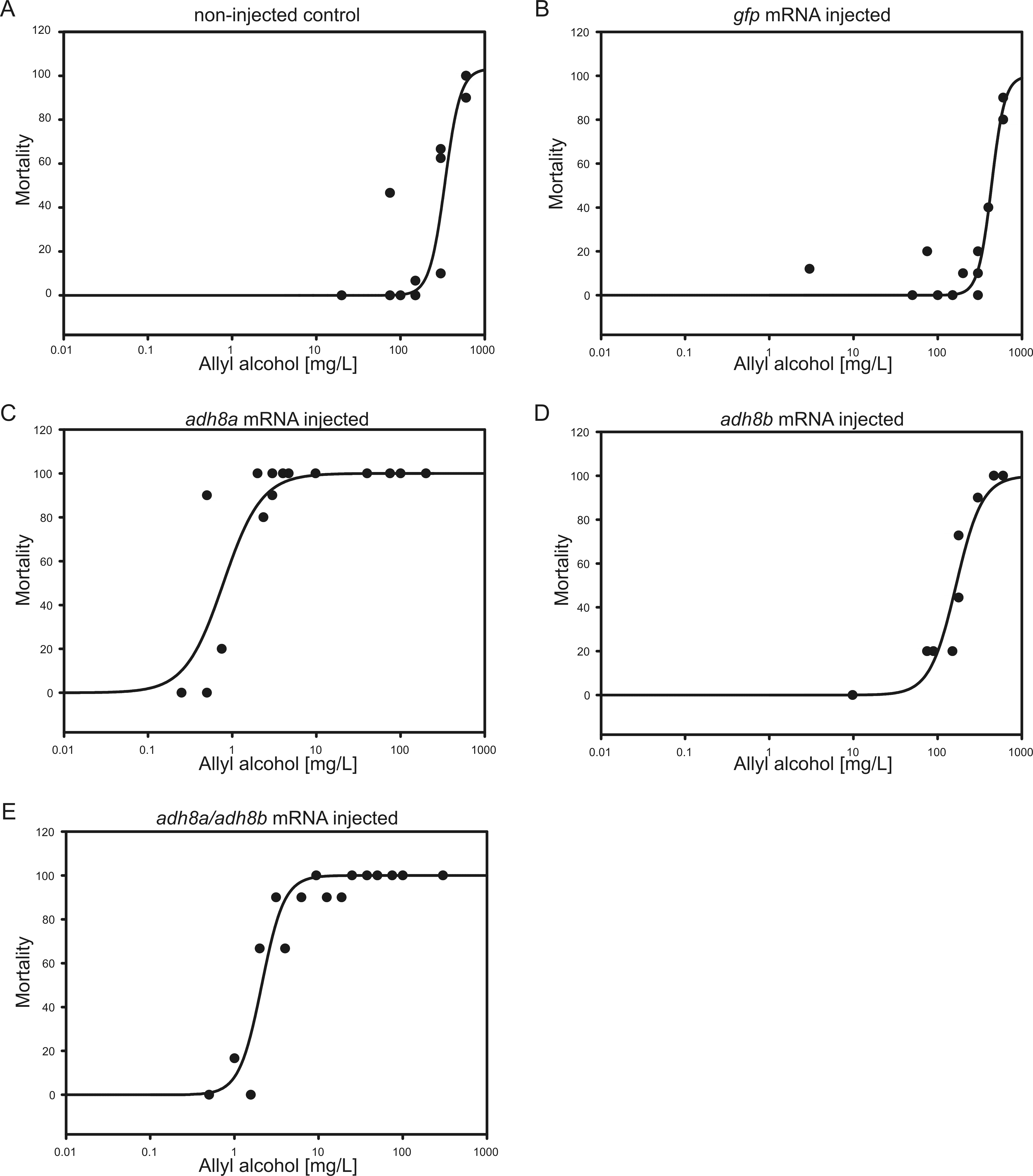

Supplement: Figure S3 — Allyl alcohol concentration-response curves of gfp , adh8a , and adh8b mRNA injected and non-injected zebrafish embryos. Allyl alcohol exposure was performed from 8-56 hpf and mortality was measured (n = 3, see also Table S1). A. non-injected (control). B. gfp mRNA injected (injection control) C. adh8a mRNA injected. D. adh8b mRNA injected. E. adh8a/adh8b mRNA injected. (TIF) [file pone.0090619.s003.tif]

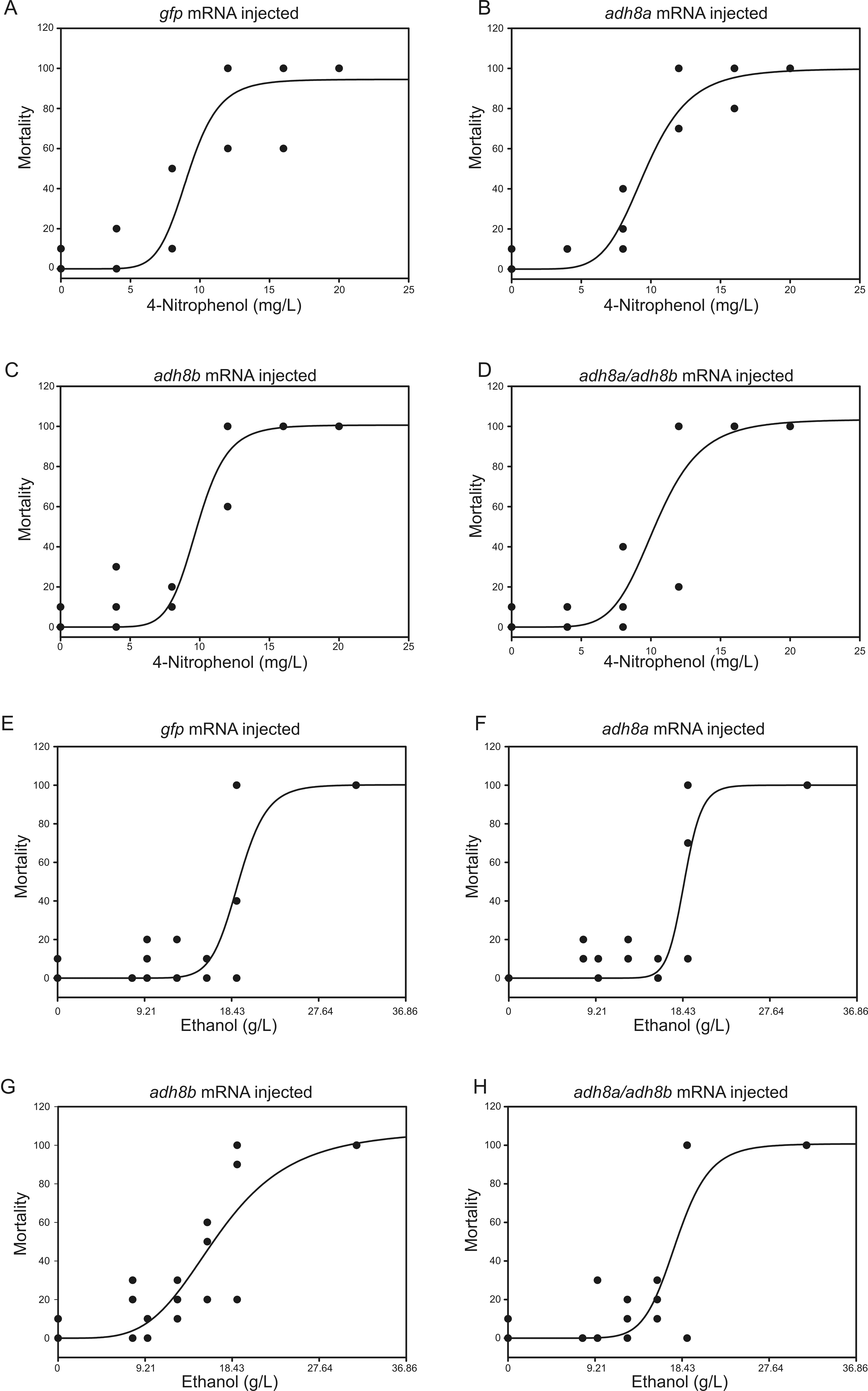

Supplement: Figure S4 — 4-Nitrophenol and ethanol concentration-response curves of gfp , adh8a , and adh8b mRNA injected zebrafish embryos. Exposures were performed in crystallization glass dishes with 10-20 embryos per vial and 30 ml of exposure medium. (A-D) 4-Nitrophenol concentrations are given in mg/L. A. gfp mRNA injected (injection control) B. adh8a mRNA injected. C. adh8b mRNA injected. D. adh8a/adh8b mRNA injected. (E-H) Ethanol concentrations are given in mM. E. gfp mRNA injected (injection control) F. adh8a mRNA injected. G. adh8b mRNA injected. H. adh8a/adh8b mRNA injected. (TIF) [file pone.0090619.s004.tif]
